# The Improved Particle Swarm Optimization Method: An Efficient Parameter Tuning Method with the Tuning Parameters of a Dual-Motor Active Disturbance Rejection Controller

**DOI:** 10.3390/s23208605

**Published:** 2023-10-20

**Authors:** Yi Deng, Jiying Zhu, Hai Liu

**Affiliations:** 1 School of Electronic and Electrical Engineering, Wuhan Textile University, Wuhan 430200, China; 2 State Key Laboratory of New Textile Materials and Advanced Processing Technologies, Wuhan Textile University, Wuhan 430200, China; 3 Faculty of Artificial Intelligence in Education, Central China Normal University, 152 Luoyu Road, Wuhan 430079, China

**Keywords:** dual-motor coupling, IPM, ADRC, permanent magnet synchronous motor (PMSM)

## Abstract

Dual-motor control systems require high synchronization maintenance. Active disturbance rejection controllers (ADRC), renowned for their exceptional immunity to interference, rapid response time, and robustness, have gained widespread adoption as a prominent control strategy. The stability of the dual-motor system can be enhanced by employing an ADRC. However, setting ADRC parameters is challenging. This paper proposes an improved particle swarm optimization method (IPM) to alleviate the difficulty in parameter setting. We initially developed a simplified dual-motor ADRC model that includes current loop and speed loop ADRCs. Furthermore, aiming at maintaining synchronization of the dual-motor control system, the simplified dual-motor ADRC model and IPM method are combined. The experimental results demonstrate that in comparison with state-of-the-art methods, the proposed optimized dual-motor ADRC exhibits superior robustness, minimal overshoots, negligible steady-state errors, and high stability.

## 1. Introduction

Recently, multi-motor coordinated synchronization control has been extensively employed in various industrial sectors, such as wind energy generation, industrial robotics, electric vehicles, and material handling [1]. Demands for the coordinated synchronization control precision of motors are rising, together with the repeated upgrading of industrial output [2]. Thus, the study of the synchronous control of multi-motor synchronous motor systems with complicated uncertainties and disturbances is urgently needed.

Dual-motor synchronous control requires that the two motors must maintain the same speed in the working state. In order to improve the synchronization performance of dual-motor system, a parallel control strategy was proposed. However, without feedback of the speed difference of the two motors, its synchronization control is poor [3]. The master–slave control strategy takes the output speed of the master motor as the reference speed of the slave motor. However, without feedback, the synchronization performance of master–slave control is not satisfactory [4]. The structure of deviation coupling control and circular coupling control is complex, and the amount of calculation is large, which is not conducive to practical application [5,6]. A cross-coupling control strategy was proposed by Professor Koren to realize the synchronous and coordinated control of dual motors in 1980, and the cross-coupling structure became a common dual-motor coupling structure [7,8].

With the advantages of simple structure and easy implementation, PI control is widely used for motor control. Han first proposed the active disturbance rejection controller (ADRC) [9]; with its advantages of strong stability and high precision, it became a better alternative to PI control [10]. Nevertheless, the complexity of parameter selection in ADRCs is quite substantial, and these selections inevitably affect the control performance of the system. Therefore, the choice of ADRC parameters is an important problem. At present, methods for tuning ADRC parameters mainly include [11] empirical methods, bandwidth methods (BMs), time-scale tuning methods, and intelligent algorithms.

Chen et al. proposed a data-driven iterative tuning method for a delayed ADRC, which balanced system performance and robustness through empirical relationships. The simulation results confirmed the effectiveness of this approach [12]. Wang et al. developed adjustment equations for second-order linear active interference suppression control parameters using internal model control. Their approach achieved satisfactory control performance for oscillation systems by minimizing the integral of the mean square error index [13]. Lu et al. introduced a parameterized fuzzy self-tuning approach for a load-adaptive double-loop drive system based on an enhanced position velocity-integrated ADRC. They performed experimental tests using a selective compliant assembly robot arm, which demonstrated exceptional positioning accuracy, rapid response time, and robust adaptability to changes in the applied load [14]. However, empirical methods are overly reliant on expert experience and may not yield optimal solutions.

Gao et al. proposed a controller parameterization approach by analyzing Han’s ADRC controller [9], wherein the controller parameters were expressed as a function of loop gain bandwidth. This methodology simplified the parameter adjustment process [15]. Qu et al. introduced a linear–nonlinear switching ADRC strategy, in which the extended state observer (ESO) and the state error feedback (SEF) control law adopted this linear–nonlinear switch. The experimental results demonstrated that following parameter adjustment using the BM, this approach exhibited enhanced resistance to disturbances and held promising potential for practical applications in engineering [16]. Zhang et al. built upon the foundation established by Gao [15] in summarizing the effects of various parameters on system dynamics and adjusted gain parameters of ESO using a BM. The experimental results under different operating conditions demonstrated that the anti-interference performance of grid-connected inverters can be effectively enhanced by nonlinear ADRCs (NLADRCs) with adjusted parameters [17]. Nevertheless, the BM is highly sensitive to small changes in control system parameters, which may cause instability in some cases. Moreover, it is usually suitable for linear or nearly linear systems but may not provide satisfactory control performance for highly nonlinear systems with complex coupling and cross-effects.

Li et al. proposed a time-scale-based parameter adjustment method for ADRCs, which facilitated the parameter tuning process [18]. Similarly, Shao et al. developed an effective approach to adjust the parameters of motion-induced ADRC by considering the relationship between time scale and multiple parameters [19]. However, this method relies on accurate interference prediction and may not perform well if the interference characteristics change. It also involves relatively complex calculations.

Yang et al. utilized a particle swarm optimization (PSO) algorithm to refine the pertinent parameters within the initial ADRC parameter configuration approach, consequently attaining accurate motion control of the antenna servo system. The simulation result indicated that the enhanced ADRC exhibited advantages such as minimal overshoot, rapid response, strong anti-interference capability, high reliability, and robustness [20]. Ma et al. proposed integrating the ADRC with intelligent algorithms to enhance its controller performance by introducing fuzzy inference and radial basis function neural network algorithms for self-adjustment of controller parameters. The experimental results indicated that this approach provided a reliable guarantee for interventional surgical robot stability [21]. Liu et al. introduced a novel ADRC design based on an improved meme algorithm (IMA) for permanent magnet synchronous motors (PMSMs). The experimental findings revealed significant optimization effects of the IMA-based ADRC [22]. Moreover, various methods exist for tuning ADRC parameters, including differential evolution algorithms [23,24], artificial bee colonies [25], genetic algorithms [26], whale optimization algorithms [27,28], gray wolves [29,30,31,32], neural network algorithms [21,33,34,35,36], and other sophisticated approaches. Nevertheless, intelligent algorithms may be prone to premature convergence, leading to suboptimal parameter selection. The ADRC parameter adjustment methods are summarized in Table 1.

In this work, an improved PSO method (IPM) is proposed to solve the parameter-tuning problem in ADRCs. The key findings of this article can be succinctly summarized as follows:

(i) The proposed method effectively addresses the issue of ADRC parameter setting without local premature convergence and does not rely on dataset training.
(ii) The ADRC is added into the dual-motor coupling control system to replace the PI control in the traditional dual-motor coupling control system. The new dual-motor coupling control system uses the ADRC speed loop and current loop as feedback control.
(iii) The experimental results demonstrate the efficient parameter selection capability of the IPM for the ADRC, leading to reduced overshoot steady-state errors (REs) and enhanced immunity performance in dual-motor ADRC systems. The evaluation function (EF) designed in this study is only 1.139, which is 1.298 lower than that of the state-of-the-art method.

The remainder of this paper is structured as follows. A mathematical model of PMSM is provided in Section 2. A dual-motor system is integrated with the IPM to form a driver in Section 3, and each part of the drive is introduced in detail, including the motor control mode, ADRC selection, and IPM process and parameter selection. Comparison experiment is used to verify that the IPM has better performance in ADRC parameter setting in Section 4. The content of the paper is summarized in Section 5.

## 2. Mathematical Model of SPMSM

The subject of investigation in this paper is the surface-mounted PMSM (SPMSM), ignoring the nonlinear properties of the PMSM, the air gap permeance, the internal permeance of the permanent magnet, and the winding damping on the rotor. Moreover, the three-phase stator winding of the motor is symmetrically distributed in space, and the air gap magnetic field is sinusoidally distributed. The mathematical model of the SPMSM can be acquired in accordance with the motor control theory in [37].

The stator voltage equation of the PMSM in the *d–q* synchronous rotating coordinate system is

(1) $$\left\{\begin{array}{l} u_{d}=Ri_{d}+L_{d}\frac{d}{dt}i_{d}-\omega_{e}L_{q}i_{q}\\ u_{q}=Ri_{q}+L_{q}\frac{d}{dt}i_{q}+\omega_{e}\left(L_{d}i_{d}+\Psi_{f}\right) \end{array}\right.,$$

where $u_{d}$ and $u_{q}$ are the stator voltage components of *d*- and *q*-axes, respectively; $\Psi_{d}$ and $\Psi_{q}$ are the stator flux components of *d*- and *q*-axes, respectively; $L_{d}$ and $L_{q}$ are the inductance components of *d*- and *q*-axes, respectively; $\omega_{e}$ is the electric angular velocity; and $\;\Psi_{f}$ is permanent-magnet flux linkage.

The PMSM electromagnetic torque equation is

(2) $$T_{e}=\frac{3}{2}p_{n}i_{q}\left[i_{d}\left(L_{d}-L_{q}\right)+\Psi_{f}\right].$$

The SPMSM’s permanent magnet has a homogeneous air gap, and its relative permeability is essentially 1 when $L_{d}=L_{q}$. The electromagnetic torque may be expressed as follows:

(3) $$T_{e}=\frac{3}{2}p_{n}\Psi_{f}i_{q},$$

where $T_{e}$ is the PMSM electromagnetic torque and $p_{n}$ is the number of pole pairs.

According to the force’s equilibrium conditions, the mechanical motion equation of the SPMSM is as follows:

(4) $$\frac{J}{p_{n}}\frac{d\omega }{dt}=T_{e}-T_{l}-B\frac{\omega }{p_{n}},$$

where J is the moment of inertia, B is the damping coefficient, $T_{l}$ is the load torque, and ω is the mechanical angular velocity of the motor.

## 3. Proposed IPM

**Figure 1:**
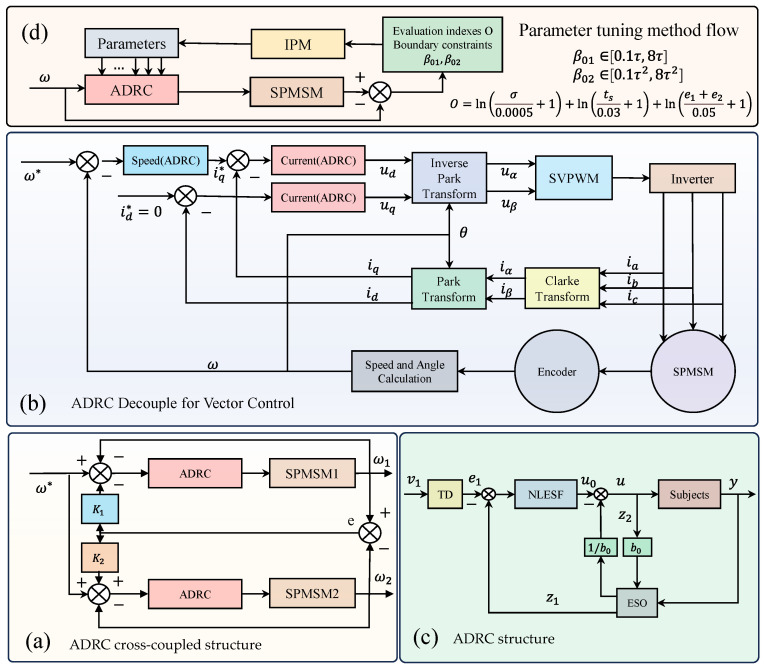
Specific components of the driver: (**a**) ADRC cross-coupled structure; (**b**) ADRC vector control; (**c**) ADRC structure; (**d**) flow of IPM.

In this paper, ADRC parameters are optimized using the IPM. For ease of use, the dual-motor system is integrated with the IPM to form a driver for optimizing dual-motor system parameters. The specific components of the driver are illustrated in Figure 1, namely (a) the ADRC cross-coupled structure, (b) the ADRC vector control (in order to reflect the anti-interference performance of ADRC, we set the compensation coefficients $K_{1}$ and $K_{2}$ to 1), (c) the ADRC structure, and (d) the flow of the IPM. Each module will be described in detail subsequently.

### 3.1. ADRC Decouping for Vector Control

According to Equation (2), the electromagnetic torque $T_{e}$ is directly proportional to the amplitude of the stator current $i_{q},$ whereas the excitation flux chain $\Psi_{f}$ remains constant. In this paper, $i_{d}\;$= 0 control is used to achieve the vector control of the motor. At this time, the stator current vector only has a q-axis component, and the stator magnetomotive force vector is perpendicular to the rotor permanent magnet field vector. Therefore, the electromagnetic torque can be controlled directly through the control of $i_{q}$. The speed of the motor under the electromagnetic torque can be obtained by Equation (4).

The following is a brief description of the vector control process. The sensor detects the value of the PMSM stator current. Clarke transformation and Park transformation are used to complete the conversion of the stator current, from a natural coordinate system to a *d–q* coordinate system. The error value between the obtained current value and the given value of the current loop is subsequently utilized as the input for the current ADRC, enabling acquisition of the desired stator voltage value in the *d–q* coordinate system. Finally, the actual stator voltage value is ultimately obtained by means of the inverse coordinate transformation, which serves as the input for driving the motor through space vector pulse width modulation. The vector control system block diagram of the PMSM is depicted in Figure 1b.

### 3.2. Design and Implementation of ADRC

#### 3.2.1. Composition of ADRC

The ADRC approach does not rely on a precise system model and does not necessitate any specific information regarding the disturbance. It exhibits the capability to achieve control for nonlinear uncertain systems. The core components of the ADRC primarily comprise the tracking differentiator (TD), extended state observer (ESO), and state error feedback (SEF) control law, as illustrated in Figure 1c.

To promptly monitor the input signal and estimate its approximate derivative, the ADRC utilizes a nonlinear TD, along with the principle of quickest control. Simultaneously, the transition process for the input signal is established, where the speed factor *r* determines how rapidly this change occurs. The discrete form of the quickest TD can be mathematically formulated as follows:

(5) $$\left\{\begin{array}{l} fh=fhan(v_{1}(k)-v,v_{2}(k),r,h_{0})\\ v_{1}(k+1)=v_{1}(k)+hv_{2}(k)\\ v_{2}(k+1)=v_{2}(k)+hfh \end{array}\right.,$$

where v is the input signal of the controller; $v_{1}(k)$ is the tracking signal of v; $v_{2}(k)$ is the approximate differential of v; $h_{0}$ is the filtering factor; h is the integral step length; and fhan is the fastest control function, and its expression is

(6) $$\left\{\begin{array}{l} d=rh\\ d_{0}=hd\\ y=v_{1}+hv_{2}\\ a_{0}=\sqrt{d^{2}+8r\left|y\right|}\\ a=\left\{\begin{array}{l} v_{2}+\frac{\left(a_{0}-d\right)}{2},\left|y\right|>d_{0}\\ v_{2}+\frac{y}{h},\left|y\right|\leq d_{0} \end{array}\right.\\ fhan=-\left\{\begin{array}{l} rsgn\left(a\right),\left|a\right|>d\\ r\frac{a}{d},\left|a\right|\leq d \end{array}\right. \end{array}\right.,$$

where $sgn\left(\cdot \right)$ is the sign function.

The ESO effectively mitigates internal and external disturbances of the system by incorporating known and unknown dynamics as a state variable, estimating real-time observations, and adding compensation. In the case of a second-order nonlinear system, the nonlinear ESO (NLESO) can be expressed as follows:

(7) $$\left\{\begin{array}{l} e=z_{1}-y\\ \dot{z_{1}}=z_{2}-\beta_{01}fal(e,\alpha_{1},\delta )\\ \dot{z_{2}}=z_{3}-\beta_{02}fal\left(e,\alpha_{2},\delta \right)+bu\\ \dot{z_{3}}=-\beta_{03}fal\left(e,\alpha_{3},\delta \right) \end{array}\;\right.,$$

where the fal(·) function is a continuous power function with a line segment near the origin, which can be expressed as

(8) $$fal\left(e,\alpha ,\delta \right)=\left\{\begin{array}{l} {\left|e\right|}^{\alpha }sgn\left(e\right),\left|e\right|>\delta \\ e/\delta^{\left(1-\alpha \right)},\left|e\right|\leq \delta \end{array}\right..$$

The nonlinear SEF (NLSEF) control law is expressed as follows:

(9) $$\left\{\begin{array}{l} e_{1}=v_{1}-z_{1}\\ e_{2}=v_{2}-z_{2}\\ u_{0}=k_{1}fal\left(e_{1},\alpha_{1},\delta \right)+k_{2}fal\left(e_{2},\alpha_{2},\delta \right) \end{array}\right.,$$

where $k_{1}$ and $k_{2}$ are the feedback gain coefficients.

#### 3.2.2. Design of Current Loop ADRC

The current loop is feedback to the error value between the obtained current value and the given value of the current loop, which controls the torque, thereby indirectly controlling the speed. Through the mathematical model analysis of the PMSM in Section 2, the current equation of the PMSM in the *d–q* coordinate system is

(10) $$\left\{\begin{array}{l} \frac{di_{d}}{dt}=-\frac{R}{L_{d}}i_{d}+\frac{L_{q}}{L_{d}}\omega_{e}i_{q}+\frac{1}{L_{d}}u_{d}\\ \frac{di_{q}}{dt}=-\frac{R}{L_{d}}i_{d}-\frac{1}{L_{q}}\omega_{e}(L_{d}i_{d}+\Psi_{f})+\frac{1}{L_{q}}u_{q} \end{array}\right..$$

**Figure 2:**
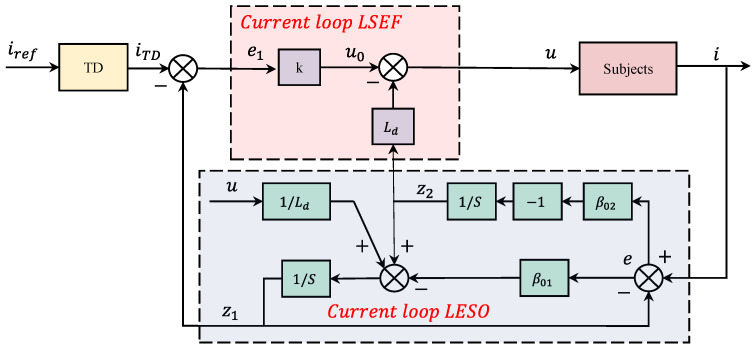
Current loop ADRC structural diagram.

According to Equation (10), the current ADRC for the d- and q-axes of motor excitation and electromagnetic torque control is designed. To achieve decoupling and simplify the controller design, feedforward compensation is required for unknown disturbances arising from back potential and coupling effects. These disturbances are considered unknown perturbations and are compensated by observing them along with external disturbances using the ESO. The current loop is controlled by a first-order ADRC structure, as depicted in Figure 2.

In this case, the corresponding first-order linear ADRC equation is as follows.

The TD module can be represented as

(11) $$\;\left\{\begin{array}{l} fh=fhan(e,r,h_{0})\\ v_{1}(k+1)=v_{1}(k)+hfh \end{array}\right..$$

The ESO module can be expressed as

(12) $$\;\;\;\left\{\begin{array}{l} e=z_{1}-y\\ \dot{z_{1}}=z_{2}-\beta_{01}e+bu\\ \dot{z_{2}}=-\beta_{02}e \end{array}\right..$$

The linear (SEF) LSEF module is

(13) $$\left\{\begin{array}{l} e=v_{1}-z_{1}\\ u_{0}=k\cdot e_{1}\\ u=u_{0}-\frac{z_{2}}{b_{0}} \end{array}\right..$$

#### 3.2.3. Design of Speed Loop ADRC

The speed loop is feedback to the error value between set speed and actual speed, which directly control the motor speed. The speed equation of the motor can be obtained as

(14) $$\frac{d\omega }{dt}=-\frac{T_{l}}{J}-\frac{B\omega }{J}+\frac{p_{n}\Psi_{f}}{J}i_{q}.$$

The motor’s different operating conditions, load torque $T_{l}$, and moment of inertia J can cause unknown disturbances that significantly affect speed performance due to their wide range of variation. Similar to the current loop design, these disturbances can be treated as unknowns and compensated for in real time using ESO estimates. The speed loop control incorporates a first-order NLADRC, and an LSEF control law is employed for parameter tuning. This selection is based on the fact that the disparity in the system performance between NLSEF and LSEF control laws is negligible. The structure of the speed loop ADRC mirrors that of the current loop, with the corresponding first-order NLADRC equation being the following:

The TD module and LSEF modules are the same as the first-order linear ADRC, and the NLESO module can be represented as

(15) $$\;\;\left\{\begin{array}{l} e=z_{1}-y\\ \dot{z_{1}}=z_{2}-\beta_{01}fal(e,\alpha_{1},\delta )\\ \dot{z_{2}}=-\beta_{02}fal\left(e,\alpha_{2},\delta \right) \end{array}\right..$$

#### 3.2.4. Stability of Extended State Observer

In order to verify the stability of the ESO, we make some assumptions [9].

**Assumption** **1.** $f\left(x_{1},x_{2}\right)=\omega_{0}=const$ *and* α=0.5. *The object is:*

(16) $$\left\{\begin{array}{l} \dot{x_{1}}=x_{2}\\ \dot{x_{2}}=\omega_{0}+bu\\ y=x_{1} \end{array}\right..$$

The Lyapunov function of the following form:

(17) $$V\left(e_{1},e_{2}\right)=A{\left|e_{1}\right|}^{\frac{3}{2}}-Be_{1}e_{2}+C{e_{2}}^{2}.$$

Equation (17) takes the derivative of both sides:

(18) $$\dot{V}=\frac{\partial V}{\partial e_{1}}\dot{e_{1}}+\frac{\partial V}{\partial e_{2}}\dot{e_{2}}=\left(\frac{3}{2}A-2C\beta_{01}-B\beta_{02}+B\beta_{01}{\left|e_{1}\right|}^{\frac{1}{2}}\right){\left|e_{1}\right|}^{-\frac{1}{4}}{\left|e_{1}\right|}^{\frac{3}{4}}sign\left(e_{1}\right)e_{2}-\;B{e_{2}}^{2}-\left(\frac{3}{2}A\beta_{01}-B\beta_{02}\right){\left|e_{1}\right|}^{2\left(\frac{3}{4}\right)}+\left(B\omega_{0}e_{1}+2C\omega_{0}e_{2}\right),$$

This expression can be viewed as a quadratic formal function of the variables ${\left|e_{1}\right|}^{\frac{3}{4}}sign\left(e_{1}\right)e_{2}$ and $e_{2}$.

(19) $$\dot{V}=-X{\left|e_{1}\right|}^{2\left(\frac{3}{4}\right)}+Y{\left|e_{1}\right|}^{\frac{3}{4}}sign\left(e_{1}\right)e_{2}-Z{e_{2}}^{2}+\left(B\omega_{0}e_{1}+2C\omega_{0}e_{2}\right).$$

where $X=\frac{3}{2}A\beta_{01}-B\beta_{02},Y=\left(\frac{3}{2}A-2C\beta_{01}-B\beta_{02}+B\beta_{01}{\left|e_{1}\right|}^{\frac{1}{2}}\right){\left|e_{1}\right|}^{-\frac{1}{4}},Z=B$.

The necessary and sufficient conditions for the quadratic part of this quadratic function to be negative definite are $X>0,\;Y>0,\;Z>0,\;Y^{2}-4XZ<0.$ In order to satisfy this condition, we can derive $B>0,\;3\frac{A}{B}>2\frac{\beta_{01}}{\beta_{02}},\;3A>4C\beta_{02}$.

According to the above solution, $\beta_{01}$ and $\beta_{02}$ are given, and the values of *A*, *B*, and *C* can be obtained. In this case, the Lyapunov function is positive definite and there exists suitable *A*, *B*, and *C* to ensure that Equation (19) satisfies the negative definite condition, which satisfies Lyapunov’s stability theorem.

### 3.3. IPM

In comparison with the conventional proportional–integral–derivative controller, the ADRC requires adjustment of a greater number of parameters. Once the structure is determined, the selection of ADRC parameters directly affects the system control performance, necessitating the design of an algorithm for parameter tuning. Subsequently, this paper will provide a detailed introduction to the parameter-tuning process using the proposed IPM.

#### 3.3.1. Tuning of ADRC Parameters Using IPM

**Figure 3:**
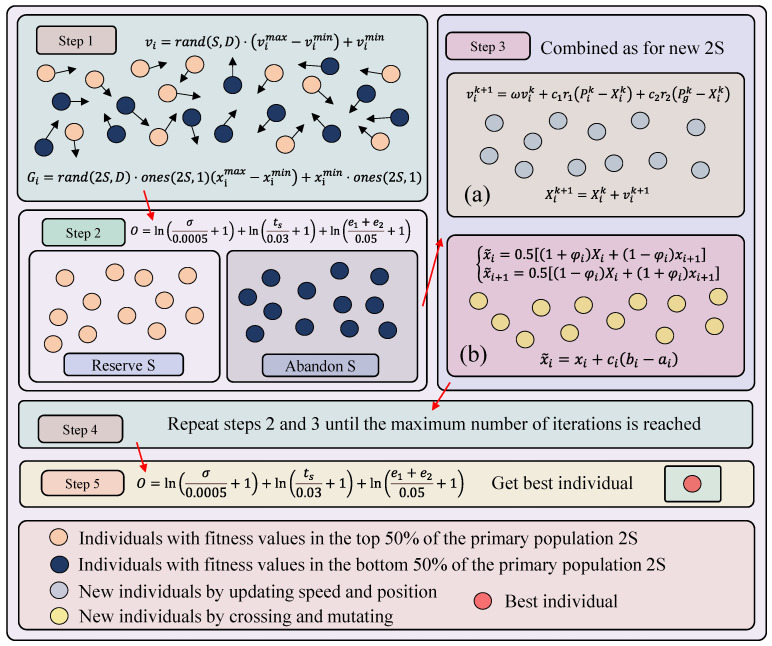
Overall procedure of IPM method.

The two parameters $\beta_{01}$ and $\beta_{02}$ of the ESO in the speed loop ADRC directly affect the system’s state estimation, particularly the precision of the total disturbance. However, the NLESO parameters have no defined range and must be adjusted continuously to be set. In this work, an IPM is proposed to adjust the ADRC’s two parameters $\beta_{01}$ and $\beta_{02}$. The specific procedure of the optimization method is illustrated in Figure 3.

The IPM method described in this research mostly consists of the next five steps.

Step 1: Initializing the particle individual and population to generate the initial population. Initializing the particle velocity; the process is

(20) $$G_{i}=\mathrm{rand}\left(2S,D\right)\cdot ones(2S,1){(x}_{i}^{max}-x_{i}^{min})+x_{i}^{min}\cdot ones(2S,1).$$

where $rand\left(A,B\right)$ is a random matrix with row *A* and column *B*, and [0, 1] is the range of values for each member. D is the dimension; $x_{i}^{max}$ and $x_{i}^{min}$ are the upper and lower limits of the individual. ones(A,B) is a constant matrix with row *A* and column *B*, whose element values are all 1; and *S* is the population size.

(21) $$v_{i}=rand\left(S,D\right)\cdot \left(v_{i}^{max}-v_{i}^{min}\right)+v_{i}^{min}.$$

where $v_{i}^{max}$ and $v_{i}^{min}$ are the maximum and minimum values of particle velocity.

Step 2: The fitness of the original particle swarm is determined, as the objective of parameter optimization in this paper is to enhance the control performance of the dual-SPMSM synchronous system. The evaluation criterion for the control parameters is based on the output performance of the system. A control system’s performance can typically be assessed in terms of speed, stability, and accuracy.

To evaluate the parameters obtained by the IPM accurately, this paper constructs a new evaluation function (EF). The primary indications to be considered for the control needs of the dual-PMSM synchronous system are the following:

(1) The adjustment time (AT) $t_{s}$ should be considered to reach the target speed as quickly as possible.
(2) In the process of motor acceleration, when the motor exceeds the set speed, the difference should be as small as possible, which means the system overshoot σ should be small.
(3) The fluctuation between each motor and the set speed should be as small as possible.

To measure the variation after the steady state, the absolute value of the motor speed and setpoint over a period of time after the steady state is chosen as an integral, which may be written as follows:

(22) $$\left\{\begin{array}{l} e_{1}=\int_{t_{1}}^{t}\left|\omega_{s}-\omega_{1}\right|dt\\ e_{2}=\int_{t_{1}}^{t}\left|\omega_{s}-\omega_{2}\right|dt \end{array}\right..$$

where $t_{1}$ is the estimated fluctuation time, and t is the overall length of the simulation. In this paper, the overshoot is typically less than 0.05%, the correction time is 0.03 s, and the fluctuation is 0.05.

The fitness assessment function may be stated as follows using the methodology presented above:

(23) $$O=\ln\left(\frac{\sigma }{0.0005}+1\right)+\ln\left(\frac{t_{s}}{0.03}+1\right)+\ln\left(\frac{e_{1}+e_{2}}{0.05}+1\right).$$

Step 3: Following the fitness calculation, each individual is ranked by fitness from large to small, and the *S* individuals with the highest fitness are selected. To create a new particle swarm $S_{1}$, each iteration modifies the particle’s location and velocity. The mechanism of updating is

(24) $$v_{i}^{k+1}=\omega v_{i}^{k}+c_{1}r_{1}\left(P_{i}^{k}-X_{i}^{k}\right)+c_{2}r_{2}\left(P_{g}^{k}-X_{i}^{k}\right),$$

(25) $$x_{i}^{k+1}=x_{i}^{k}+v_{i}^{k+1}.$$

where ω is the inertia weight; k is the number of current iterations; and the nonnegative constants $c_{1}$ and $c_{2}$ represent individual and group learning factors. $r_{1}$ and $r_{2}$ are two random numbers in the [0, 1] range. $P_{i}$ and $P_{g}$ are the extreme value positions of individual fitness and population fitness.

(26) $$\left\{\begin{array}{l} {\tilde{x}}_{i}=0.5\times [\left(1+\varphi_{i}\right)x_{i}+(1-\varphi_{i})x_{i+1}]\\ {\tilde{x}}_{i+1}=0.5\times \left[\left(1-\varphi_{i}\right)x_{i}+\left(1+\varphi_{i}\right)x_{i+1}\right] \end{array}\right.,$$

where ${\tilde{x}}_{i}$ and ${\tilde{x}}_{i+1}$ are the new generation of population individuals after crossover operation, $x_{i}$ and $x_{i+1}$ are the original population individuals, and $\varphi_{i}$ can be expressed as

(27) $$\varphi_{i}=\left\{\begin{array}{l} {\left(2c_{i}\right)}^{\frac{1}{\eta +1}},0<c_{i}\leq 0.5\\ {\left(\frac{1}{2\left(1-c_{i}\right)}\right)}^{\frac{1}{\eta +1}},0.5<c_{i}<1 \end{array}\right..$$

where $c_{i}$ is a random number between [0, 1], and η is the distribution index.

(28) $${\tilde{X}}_{i}=X_{i}+c_{i}\left(x_{i}^{max}-x_{i}^{min}\right).$$

To obtain a new population $S_{2}$, the chosen $S_{2}$ individuals are concurrently put through crossover operation (26) and mutation operation (28). A new population 2S is created by combining the two optimal populations.

Step 4: Repeating Steps 2 and 3 as necessary to reach the predetermined maximum number of iterations, $T_{max}$.

Step 5: After the last iteration, the final population is acquired, and the fitness is computed to find the best individual.

#### 3.3.2. Parameter Selection Using IPM

The larger the population size S is, the greater the diversity of the initial population will be, thereby facilitating the attainment of an optimal solution. However, this optimization process requires more time. In this paper, a total of 100 populations are selected for analysis. The maximum number of iterations $T_{max}$ is set to 30.

(29) $$\omega \left(k\right)=\omega_{s}-\left(\omega_{s}-\omega_{e}\right)\left(\frac{k}{T_{max}}\right).$$

where $\omega_{s}$ is the initial weight and $\omega_{e}$ is the end weight.

The inertia weight ω is the embodiment of the particle’s ability to inherit the previous speed, which has a great influence on the particle search. In this paper, a dynamic change method of ω is shown in Equation (29). In the early stage, ω changes slowly and the value is large, maintaining the global search ability of the method. In the later stage, the change in ω is accelerated and the local optimization ability of the method is improved.

The individual learning factor $c_{1}$ and the group learning factor $c_{2}$ are usually in the range of [0, 2]. In this paper, the values of $c_{1}$ and $c_{2}\;$ are equal to preserve the method’s symmetry. The particle speed updates slowly, the search range is narrow, and the process is more reliable when the values of $c_{1}$ and $c_{2}$ are modest. After several experiments, the correct learning factor is determined to be 1.4995 ($c_{1}=c_{2}$). The reference BM’s ideological parameter optimization range is displayed in Table 2.

The probability of individuals intersecting during the iterative process is referred to as the crossover probability $p_{c}$. Ranging from 0.7 to 1.0, the value of crossover probability represents the likelihood of individual crossing. A smaller crossover probability may result in a more stable search process for the method but can potentially limit its exploration capabilities and yield suboptimal outcomes. The search process of the method becomes more exploratory when the crossover chance is high. However, this condition can also lead to a deceleration in convergence speed. The mutation probability $p_{m}$ represents the likelihood of each individual undergoing changes through the method’s mutation operation, and it is a real number ranging from 0.01 to 0.06. The method’s search process is more stable when the mutation chance is low, but it may also arrive at a local optimum solution. The method’s search process is more exploratory when the mutation chance is high, but it can also potentially slow down at which the solution converges.

In this paper, a function is used to acquire the optimal parameters for the IPM. The evaluation criteria encompass computational time and performance enhancement achieved through optimization. Specifically, the convergence iterations required for method T and the dual-motor system overshoot σ are considered assessment metrics. The specific expression of the designed function is as follows:

(30) $$R=\ln\left[\bar{\sigma }+\left(\frac{T}{100}\right)\right].$$

where $\bar{\sigma }$ represents the magnitude of overshoot following standard processing. The proper crossover probability is $p_{c}=0.9$, and the suitable mutation probability is $p_{m}=0.02$. The rationale behind selecting these parameter values for the method will be elucidated in Section 4, which encompasses the experimental results and subsequent discussion.

## 4. Experimental Results and Discussion

### 4.1. Experimental Platform

To enhance the verification of the proposed IPM for ADRC parameter tuning, a corresponding simulation experimental platform is constructed. Dynamic loading is applied to the PMSM as a disturbance source, whereas the predefined reference speed and torque signals are utilized for the control of motor speed. A virtual environment for controlling the speed of PMSMs is also established. In this paper, we chose two identical motors, and their respective parameters presented in Table 3.

### 4.2. Comparison of Experimental Results and Analysis

To substantiate the efficacy of the fitness function proposed in this study, a series of comprehensive performance index functions commonly employed at present, namely IAE, ISSE, ITAE, and ITSE, are utilized for comparative testing. The characteristics of each performance indicator function are presented in Table 4.

**Figure 4:**
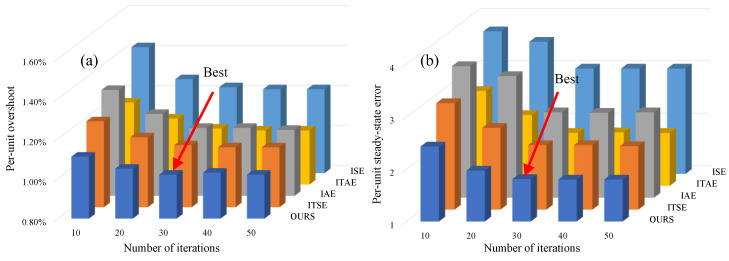
Comparison of different fitness functions under different numbers of iterations: (**a**) value of per-unit overshoot; (**b**) value of per-unit RE.

In Table 4, e(t) is the system output error and t is the time. The transient response characteristics are emphasized when the error is represented as a square. Conversely, the RE characteristics are emphasized, whereas the error exists in the form of absolute value. The steady-state performance of the system is further enhanced when the error is multiplied by time, and this enhancement becomes more pronounced with each successive power increment. Currently, the existing comprehensive EF cannot balance the transient response and RE. The comparison between the fitness function designed in this paper and these comprehensive EFs (evaluation function) is shown in Figure 4.

The comparison after standardized processing is shown in Figure 4, where the abscissa is the number of iterations, and the ordinate is different EFs. Figure 4a is the magnitude of the overshoot of various EFs after different iterations, and Figure 4b is the magnitude of the RE. In Figure 4a, the overshoot basically does not change and the IPM converges after 30 iterations; moreover, the overshoot of the fitness function proposed in this paper is the smallest. In addition, On the performance of RE, the fitness function proposed in this paper outperforms that of other fitness functions, as demonstrated in Figure 4b. After thorough analysis, the fitness function designed in this study comprehensively considers the transient response and the RE of the dual-motor system, rendering it more suitable as a fitness function for IPM.

**Figure 5:**
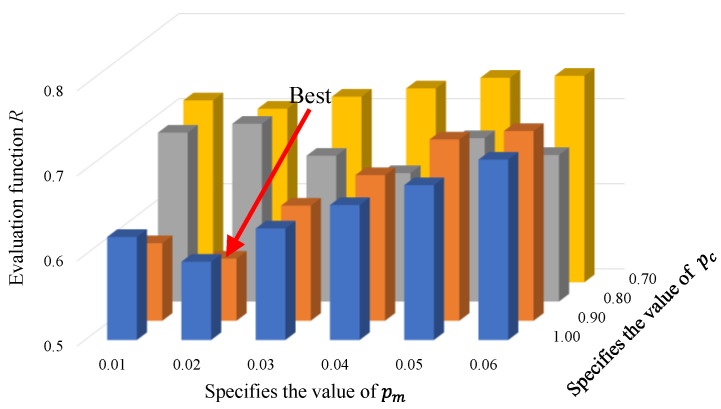
Outcome of the EF *R* under varying conditions of $p_{c}$ and $p_{m}$.

The IPM still encounters challenges in parameter selection. Subsequently, comparative experiments are conducted to determine certain parameters of the IPM. Specifically, this paper focuses on the selection experiment of two key parameters: crossover probability $p_{c}$ and mutation probability $p_{m}$, as depicted in Figure 5.

Here, the range of $p_{c}$ is [0.7, 1.0], and the range of $p_{m}$ is [0.01, 0.06]. In Figure 5, the evaluation function R reaches its minimum value when $p_{c}=0.90$ and $p_{m}=0.02$, indicating that the dual-motor system achieves a balance between improving performance and the execution time required for the IPM. Moreover, given that $p_{c}$ and $p_{m}$ vary continuously, the evaluation function R exhibits an increasing trend. Therefore, $p_{c}=0.90$ and $p_{m}=0.02$ are more appropriate for the IPM.

**Figure 6:**
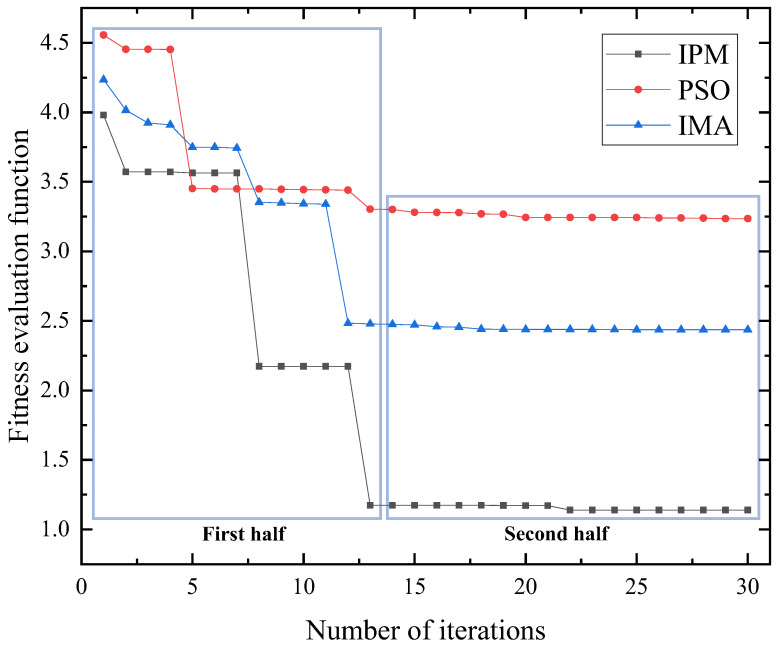
Comparison of the results of different methods.

The performance of the IPM, PSO algorithm [20], and IMA [22] are compared in the dual-motor coupling system, as depicted in Figure 6.

The convergence speed of the PSO algorithm is comparatively slower than that of the IMA and IPM, with the PSO algorithm exhibiting the most unsatisfactory performance. In addition, the IMA demonstrates a faster convergence speed compared with the IPM in the first part. The four methods in the second part exhibit convergence after approximately 15 iterations. Following convergence, the IPM demonstrates the smallest fitness function value, followed by the IMA, whereas the PSO algorithm exhibits the largest fitness function value. Moreover, in comparison to the PSO algorithm, the IPM algorithm reveals a higher resistance to being trapped in local optimal solutions due to its continuous updating of iterative populations and inertia weights. Similarly, the IMA demonstrates exceptional performance. However, it is specifically designed as a parameter-tuning algorithm for a single-PMSM ADRC, and thus lacks precision when adjusting parameters for two motors. Through the comparison and analysis of the four methods, the IPM exhibits superior performance in the proposed dual-motor system presented in this paper. It demonstrates rapid convergence speed and a reduced likelihood of converging to local optima, rendering it more suitable for parameter tuning of the dual-motor ADRC.

Moreover, the optimized nonlinear active disturbance rejection controller in the paper is compared with a linear active disturbance rejection controller; the specific results are shown in Table 5.

The control performance of the nonlinear active disturbance rejection controller is better than that of the linear active disturbance rejection controller in both the transient and steady state in Table 5.

### 4.3. Experimental Results and Analysis of the Dual-Motor System

**Figure 7:**
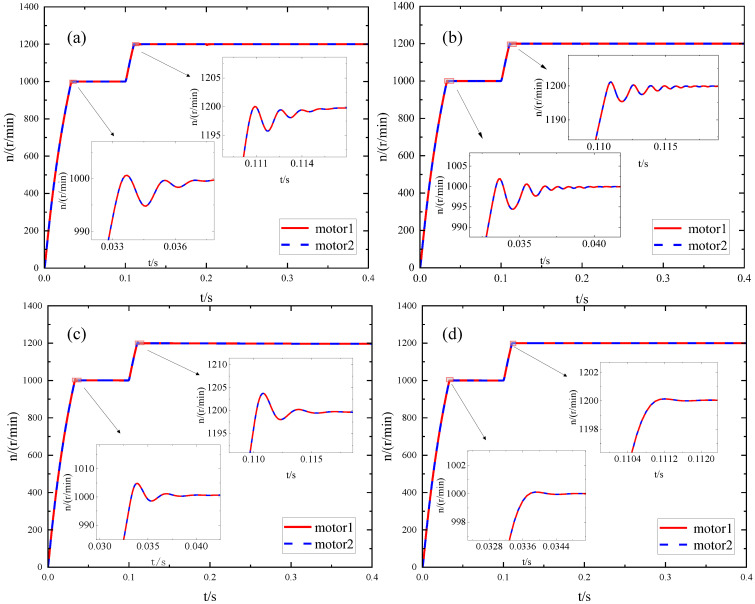
Change in no-load speed after optimization of different methods: (**a**) BM; (**b**) IMA; (**c**) PSO; (**d**) IPM.

The ADRC system parameters optimized by the BM, PSO algorithm [20], IMA optimization [22], and IPM algorithm optimization are compared and validated during motor starting, speed variation, and load addition processes to confirm the efficacy of the IPM for ADRC parameter optimization. Initially, a step signal is transmitted with an initial value of 1000 that shifts to 1200 after 0.1 s. The speed waveforms for the four methods are depicted in Figure 7a–d. The rising time (RT) among all four methods is nearly identical. However, in terms of overshoot, AT, fluctuation, and control effectiveness, all exhibiting smaller values, the IPM algorithm outperforms the other three approaches.

**Figure 8:**
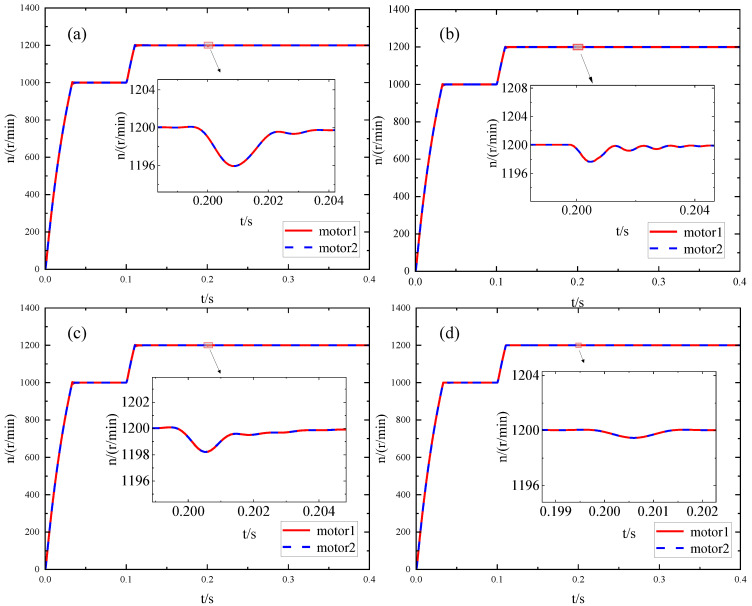
Speed change of the load motor when added after the optimization of different methods: (**a**) BM; (**b**) IMA; (**c**) PSO; (**d**) IPM.

The system is subjected to a step load of 4 N·m at 0.2 s, as illustrated in Figure 8a–d. In comparison, the IPM control exhibits superior robustness and faster recovery time for dynamic performance, and the speed fluctuation is also reduced.

**Figure 9:**
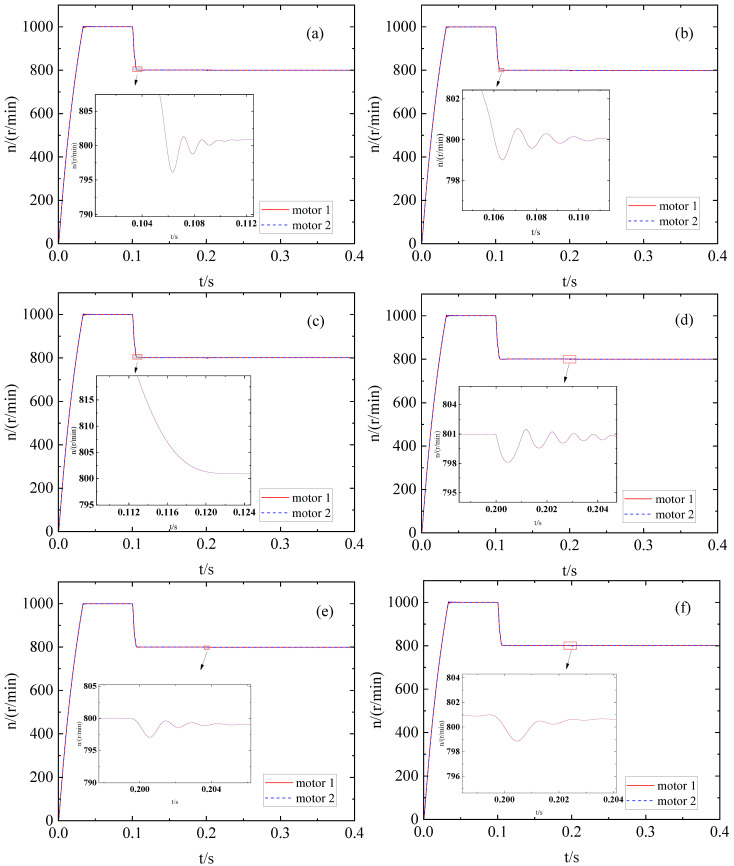
Step response downward change in no-load speed after optimization of different methods: (**a**) PSO; (**b**) IMA; (**c**) IPM. Added step load to 6 N·m change in speed after optimization of different methods: (**d**) PSO; (**e**) IMA; (**f**) IPM.

Under other conditions being equal, initially, a step signal is transmitted with an initial value of 1000 that shifts to 800 after 0.1 s and the system is subjected to a step load of 6 N·m at 0.2 s. The speed waveforms for the three methods are depicted in Figure 9a–c; moreover, the performance of the dual-motor system after loading is shown in Figure 9d,e (due to the poor performance of bandwidth method, this method is not considered when adding downward step response).

The IPM method still has basically no overshoot, and the adjustment is relatively stable, when the motor suddenly decelerates. However, the PSO and IMA methods both have oscillations. After increasing the load, although the dynamic drop of PSO is less than that of IMA, its oscillation period is too long. The IPM method can still recover quickly when the load is increased, and there is basically no oscillation.

**Figure 10:**
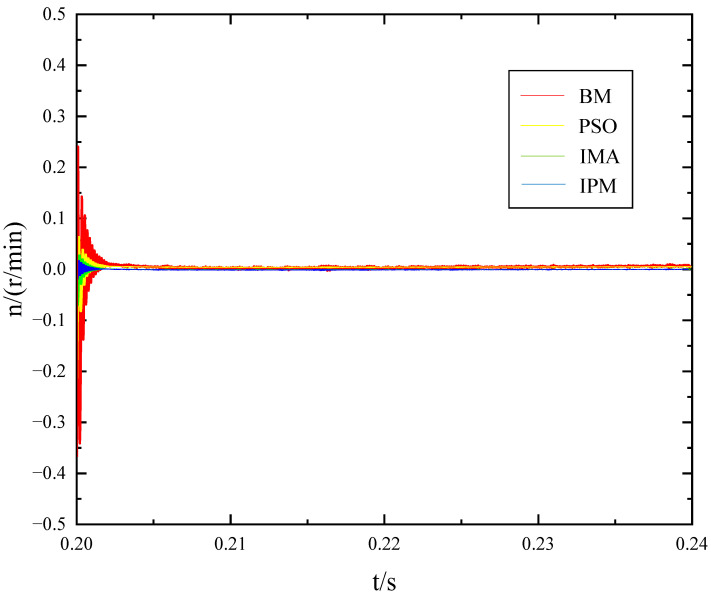
Speed difference between the two motors after the parameters are optimized by different methods.

Although two motors of the same model are selected in the simulation, deviations will exist in their parameters due to various factors, such as friction and heating during operation. Consequently, the resistance values of the experimental motors differ. Under these circumstances, when a disturbance is introduced, a speed discrepancy arises between the two motors, potentially leading to motor damage. The changes in speed difference resulting from four methods after introducing the 4 N·m disturbance are illustrated in Figure 10.

In Figure 10, the speed difference between the two motors is significantly higher than that of the other methods after adjusting the parameters using the BM. Furthermore, there may still be a slight speed difference during subsequent adjustments. However, the ADRC with the IPM proposed in this paper exhibits exceptional performance, demonstrating minimal speed difference and faster recovery, as well as enhanced stability for both motors.

### 4.4. Results and Discussion

The performance indicators (overshoot, RT, AT, RE, dynamic landing (DL), and fitness EF are the performance indices of the control system after parameter optimization, where t takes 0.1 s) of the system after employing the BM, IMA, PSO algorithm, and IPM for parameter adjustment in the dual-motor system (all data are calculated under load 4 N·m). The results are shown in Table 6.

By comparison, the ADRC system parameters tuned by the IPM exhibit smaller RE, DL, and AT than those tuned by the BM and PSO algorithm. In contrast to the IMA, a larger rise time is noted; however, other performance indicators demonstrate significant advantages.

**Figure 11:**
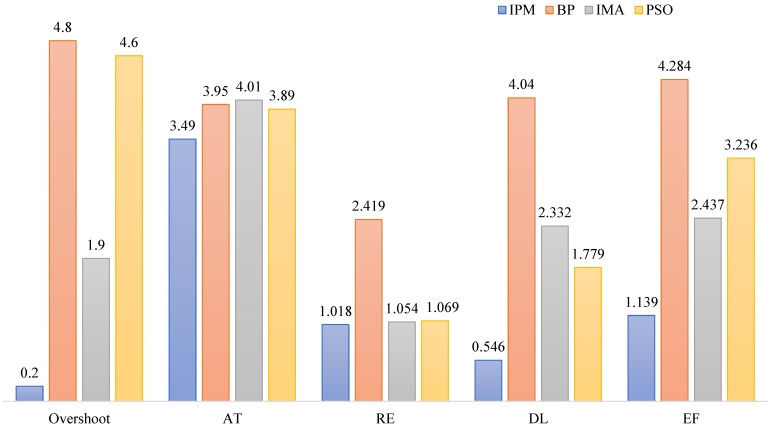
Comparison of the performance of the ADRC system after parameter optimization by different methods.

The histogram in Figure 11 represents the results of standardized processing. The image shows that the IPM algorithm exhibits superior parameter optimization, resulting in enhanced response speed, improved steady-state performance, and stronger anti-interference capabilities of the system.

## 5. Conclusions

In this paper, a parameter-tuning method based on IPM is proposed to tune the parameters of a new dual-motor coupling structure, aiming at improving the control performance of the ADRC. The present study establishes a model of a dual-motor coupled ADRC system, optimizes the parameters of the ADRC, and designs a fitness EF to assess the optimization results. In addition, the method is simulated by Matlab, verifying that the optimized parameters can significantly improve the performance of the dual-motor coupling system. The dual-motor ADRC driver based on the IPM proposed in this paper exhibits superior tracking control performance compared with the BM, PSO algorithm, and IMA method optimization. To validate the performance of the dual-motor ADRC driver proposed in this study, an experimental platform for the dual-motor system is established. The experimental results show the effectiveness of the driver. The IPM exhibits several significant performance advantages compared with the existing representative control algorithms, including enhanced response speed, reduced RE, and minimized overshoot. In the future, the incorporation of sensorless control holds the potential for cost reduction within the system. In addition, the parameter optimization method enables the dynamic adjustment of parameters online through the utilization of neural networks. The planner can dynamically adjust the controller’s parameters in real time by directly collecting on-site motor data or incorporating novel optimization algorithms, thereby enhancing the computational efficiency of the neural network [38,39,40,41]. The integration of a precise model and real-time dynamic parameter tuning represents a promising avenue for enhancing the performance of dual-motor coupled ADRC systems in future developments and it will be our next focus of research direction.

## Figures and Tables

**Table 1:** ADRC parameter adjustment methods.

Methods	Simple	Effective	Calculations Complex	Require Experience	Precocious Convergence
Empirical Method	√	×	√	√	×
Bandwidth Method	√	√	√	×	×
Time Scale Tuning	×	√	√	×	×
Intelligent Algorithm	×	√	×	×	√

**Table 2:** Optimization parameter interval setting by IPM.

Parameter	Interval
$\beta_{01}$	$\left[0.1\tau ,\;8\tau \right]$
$\beta_{02}$	$\left[0.1\tau^{2},8\tau^{2}\right]$

τ is the bandwidth of the ESO in the speed loop. The ESO bandwidth of the current and speed loop are 1200π and 60π.

**Table 3:** SPMSM parameters.

Motor Parameters	Parameter Value	Unit
Stator inductance	8.500	mH
Stator resistance	2.875	Ω
Magnetic flux linkage	0.175	Wb
Moment of inertia	0.003	kg·m^2^
Damping coefficient	0.008	N·m·s
Number of pole pairs	4	

**Table 4:** Performance index function calculation and characteristics.

Performance Index Function	Characteristics
$IAE=\int_{0}^{\infty }|e(t)|dt$	The design is simple, only the error size is considered, the RE (steady state error) is reduced, and the system accuracy is increased.
$ISE=\int_{0}^{\infty }e^{2}(t)dt$	The dominance of the transient process results in significant overshoot and REs within the system.
$ITAE=\int_{0}^{\infty }t|e(t)|dt$	With the addition of time factor, the system response speed and steady-state are better.
$ITSE=\int_{0}^{\infty }te^{2}(t)dt$	The time factor is added, the system response is fast, and the error is small.

**Table 5:** Performance of nonlinear and linear controllers.

Controllers	Overshoot	Steady-State Error
nonlinear	0.020%	0.018
linear	0.290%	0.316

**Table 6:** Performance indices after parameter optimization by different methods.

	Overshoot	RT	AT	RE	DL	EF
BM	0.4800%	0.0335	0.0395	0.8832	4.0400	4.2840
IMA	0.1900%	**0.0325**	0.0401	0.0525	2.3320	2.4370
PSO	0.4600%	0.0335	0.0389	0.0663	1.7790	3.2360
IPM	**0.0200%**	0.0339	**0.0349**	**0.0181**	**0.5460**	**1.1390**

The bold part represents the best performance.

## Data Availability

The data used to support the findings of this study are available from the corresponding author upon request.
